# 2-Methyl-5-nitro-1*H*-benzimidazol-6-amine dihydrate

**DOI:** 10.1107/S1600536811034647

**Published:** 2011-08-27

**Authors:** Sebla Dinçer, Hakan Dal, Tuncer Hökelek

**Affiliations:** a Ankara University, Department of Chemistry, 06100 Tandoğan, Ankara, Turkey; b Anadolu University, Faculty of Science, Department of Chemistry, 26470 Yenibağlar, Eskişehir, Turkey; c Hacettepe University, Department of Physics, 06800 Beytepe, Ankara, Turkey

## Abstract

The title benzimidazole mol­ecule, C_8_H_8_N_4_O_2_·2H_2_O, is planar with a maximum deviation of 0.079 (2) Å (for one of the O atoms in the nitro group). It crystallized as a dihydrate and inter­molecular O—H⋯O and N—H⋯O hydrogen bonds link the uncoordinated water mol­ecules, and the nitro and amine groups, respectively. In the crystal, N—H⋯O, O—H⋯N, O—H⋯O and C—H⋯O hydrogen bonds link the mol­ecules to form a three-dimensional network. A π–π contact between the benzene rings, [centroid–centroid distance = 3.588 (1) Å] may further stabilize the crystal structure.

## Related literature

For the anti­tumor, antihelmintic, anti­bacterial, virucidal and fungucidal properties of benzimidazole derivatives, see: Refaat (2010 ▶); Laryea *et al.* (2010 ▶); Horton *et al.* (2003 ▶); Spasov *et al.* (1999 ▶); Soula & Luu-Duc (1986 ▶). For the coord­ination and corrosion inhibitor abilities of benzimidazoles, see: Kuznetsov & Kaza­nsky (2008 ▶); Subramanyam & Mayanna (1985 ▶). For the use of benzimidazole derivatives as photographic materials and dyes, see: Hoffmann *et al.* (2011 ▶); Alamgir *et al.* (2007 ▶). For related structures, see: Hökelek *et al.* (2002 ▶); Dinçer *et al.* (2011 ▶).
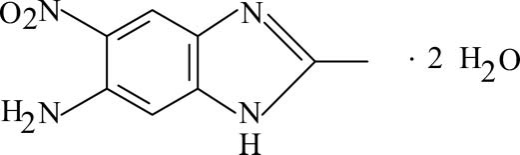


## Experimental

### 

#### Crystal data

C_8_H_8_N_4_O_2_·2H_2_O
*M* *_r_* = 228.22
Triclinic, 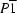

*a* = 7.0475 (3) Å
*b* = 7.2801 (3) Å
*c* = 10.9906 (4) Å
α = 76.754 (3)°
β = 71.686 (2)°
γ = 71.809 (2)°
*V* = 503.18 (4) Å^3^
*Z* = 2
Mo *K*α radiation
μ = 0.12 mm^−1^
*T* = 100 K
0.43 × 0.19 × 0.10 mm

#### Data collection

Bruker Kappa APEXII CCD area-detector diffractometer
Absorption correction: multi-scan (*SADABS*; Bruker, 2001 ▶) *T*_min_ = 0.973, *T*_max_ = 0.988
8838 measured reflections
2533 independent reflections
1800 reflections with *I* > 2σ(*I*)
*R*_int_ = 0.036

#### Refinement

*R*[*F* ^2^ > 2σ(*F* ^2^)] = 0.043
*wR*(*F* ^2^) = 0.113
*S* = 1.03
2533 reflections
174 parameters
4 restraints
H atoms treated by a mixture of independent and constrained refinement
Δρ_max_ = 0.32 e Å^−3^
Δρ_min_ = −0.31 e Å^−3^

### 

Data collection: *APEX2* (Bruker, 2007 ▶); cell refinement: *SAINT* (Bruker, 2007 ▶); data reduction: *SAINT*; program(s) used to solve structure: *SHELXS97* (Sheldrick, 2008 ▶); program(s) used to refine structure: *SHELXL97* (Sheldrick, 2008 ▶); molecular graphics: *ORTEP-3 for Windows* (Farrugia, 1997 ▶); software used to prepare material for publication: *WinGX* (Farrugia, 1999 ▶).

## Supplementary Material

Crystal structure: contains datablock(s) I, global. DOI: 10.1107/S1600536811034647/su2305sup1.cif

Structure factors: contains datablock(s) I. DOI: 10.1107/S1600536811034647/su2305Isup2.hkl

Supplementary material file. DOI: 10.1107/S1600536811034647/su2305Isup3.cml

Additional supplementary materials:  crystallographic information; 3D view; checkCIF report

## Figures and Tables

**Table 1:** Hydrogen-bond geometry (Å, °)

*D*—H⋯*A*	*D*—H	H⋯*A*	*D*⋯*A*	*D*—H⋯*A*
N4—H4⋯O3^i^	0.94 (2)	1.87 (2)	2.7735 (18)	160.4 (19)
N2—H21⋯O1^ii^	0.88 (2)	2.39 (2)	3.2212 (18)	158.8 (17)
N2—H21⋯O4^iii^	0.88 (2)	2.59 (2)	3.163 (2)	124.1 (15)
N2—H22⋯O2	0.85 (2)	2.03 (2)	2.6387 (19)	127.3 (19)
O3—H31⋯N3^iv^	0.85 (2)	1.89 (2)	2.7354 (18)	176 (2)
O3—H32⋯O4^v^	0.89 (3)	1.90 (3)	2.776 (2)	168 (3)
O4—H41⋯O3	0.90 (2)	1.88 (3)	2.7727 (19)	170 (4)
O4—H42⋯O1^vi^	0.85 (2)	2.53 (2)	3.0930 (17)	125 (2)
O4—H42⋯O2^vi^	0.85 (2)	2.17 (2)	3.0126 (17)	171 (3)
C5—H5⋯O1^ii^	0.93	2.54	3.3556 (19)	146

Symmetry codes: (i) 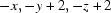; (ii) 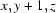; (iii) 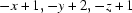; (iv) 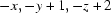; (v) 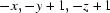; (vi) 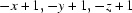.

**Fig. 1.**
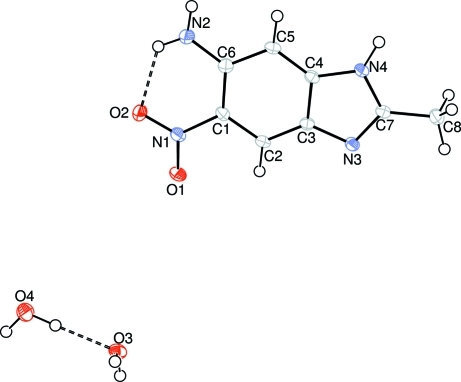
The molecular structure of the title compound, with the crystallographic labelling scheme and displacement ellipsoids drawn at the 50% probability level. The O-H···O and N-H···O hydrogen bonds are shown as dashed lines (see Table 1 for details).

**Fig. 2.**
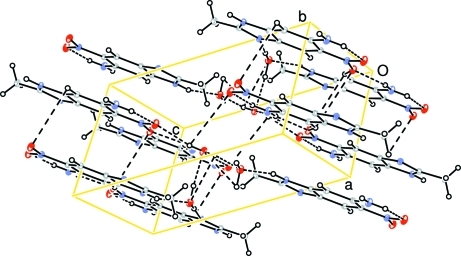
A view of the crystal packing of the title compound. Hydrogen bonds are shown as dashed lines (see Table 1 for details).

## supplementary crystallographic information

### Comment

Benzimidazole derivatives are privileged structures in pharmaceutical chemistry because of their biological activities and clinical applications. They exhibit antitumor, anthelmintic, antibacterial, virucidal and fungucidal properties (Refaat, 2010; Laryea *et al.*, 2010; Horton *et al.*, 2003; Spasov *et al.*, 1999; Soula & Luu-Duc, 1986). In addition to their biological activities, a review of the literature reveals that there are numerous studies including the coordination and corrosion inhibitor abilities of benzimidazoles (Kuznetsov & Kazansky, 2008; Subramanyam & Mayanna, 1985). Some of these derivatives, particularly nitro derivatives, are used as photographic materials in photography and on the other hand, the development of the chemistry of the benzimidazole dyes has been remarkable (Hoffmann *et al.*, 2011; Alamgir *et al.*, 2007). As a part of our ongoing investigations of benzimidazole derivatives, the title compound was synthesized and its crystal structure is reported herein.

The title molecule, (Fig. 1), consists of an imidazole ring with CH_3_, NO_2_ and NH_2_ substituents at positions 2, 5 and 6, respectively. It crystalllized with two uncoordinated water molecules. The intramolecular O—H···O and N—H···O hydrogen bonds (Table 1) link the uncoordinated water molecules and the NH_2_ and NO_2_ groups, respectively. The imidazole ring system is planar with a maximum deviation of -0.010 (2) Å (for atom C4). Atoms C8, O1, O2, N1 and N2 are 0.032 (2), 0.029 (2), -0.008 (2), -0.001 (1) and 0.079 (2) Å away from the imidazole ring mean plane, respectively.

In the crystal of the title compound N—H···O, O—H···N, O—H···O and C—H···O hydrogen bonds link the molecules to form a three-dimensional network (Table 1 and Fig. 2). The π–π contact between the benzene rings, Cg1—Cg1^i^, [symmetry code: (i) 1 - x, - y, - z, where Cg1 is the centroid of ring (C1—C6)], may further stabilize the structure, with a centroid-centroid distance of 3.588 (1) Å.

The crystal structures of similar benzimidazole derivatives, (C_7_H_4_N_4_O_4_).H_2_O (Hökelek *et al.*, 2002) and C_8_H_7_N_4_O_4_^+^. Cl^-^ (Dinçer *et al.*, 2011) have been reported.

### Experimental

For the preparation of the title compound, a solution of Na_2_S.9H_2_O (35.0 g) and S (9.0 g) in warm water (150 ml) was added slowly to a solution of 2-methyl-5,6-dinitro-1*H*-benzimidazole (30.0 g) in water (150 ml,) and the mixture was warmed at 333-343 K for 20 min. After the reaction was completed, the mixture was filtered, acidified with dilute HCl and heated until termination of H_2_S and SO_2_ formation. After cooling, the reaction mixture was treated with dilute ammonium hydroxide. The precipitate was filtered and crystallized from ethanol to give red rod-shaped crystals of the the title compound (m.p. 563-565 K).

### Refinement

Atoms H4 (of the NH group), H21 and H22 (of the NH_2_ group), H31, H32, H41 and H42 (of the water molecules) were located in a difference Fourier map and were freely refined. The C-bound H-atoms were positioned geometrically with C—H = 0.93 and 0.96 Å, for aromatic and methyl H-atoms, respectively, and constrained to ride on their parent atoms, with *U*_iso_(H) = k × *U*_eq_(C), where k = 1.5 for methyl H-atoms and k = 1.2 for all other H-atoms.

### Crystal data

**Table:**

C_8_H_8_N_4_O_2_·2H_2_O	*Z* = 2
*M**_r_* = 228.22	*F*(000) = 240
Triclinic, *P*1	*D*_x_ = 1.506 Mg m^−^^3^
Hall symbol: -P 1	Mo *K*α radiation, λ = 0.71073 Å
*a* = 7.0475 (3) Å	Cell parameters from 2308 reflections
*b* = 7.2801 (3) Å	θ = 3.0–28.1°
*c* = 10.9906 (4) Å	µ = 0.12 mm^−^^1^
α = 76.754 (3)°	*T* = 100 K
β = 71.686 (2)°	Rod-shaped, red
γ = 71.809 (2)°	0.43 × 0.19 × 0.10 mm
*V* = 503.18 (4) Å^3^	

### Data collection

**Table:**

Bruker Kappa APEXII CCD area-detector diffractometer	2533 independent reflections
Radiation source: fine-focus sealed tube	1800 reflections with *I* > 2σ(*I*)
graphite	*R*_int_ = 0.036
φ and ω scans	θ_max_ = 28.7°, θ_min_ = 2.0°
Absorption correction: multi-scan (*SADABS*; Bruker, 2001)	*h* = −9→8
*T*_min_ = 0.973, *T*_max_ = 0.988	*k* = −9→9
8838 measured reflections	*l* = −14→14

### Refinement

**Table:**

Refinement on *F*^2^	Primary atom site location: structure-invariant direct methods
Least-squares matrix: full	Secondary atom site location: difference Fourier map
*R*[*F*^2^ > 2σ(*F*^2^)] = 0.043	Hydrogen site location: inferred from neighbouring sites
*wR*(*F*^2^) = 0.113	H atoms treated by a mixture of independent and constrained refinement
*S* = 1.03	*w* = 1/[σ^2^(*F*_o_^2^) + (0.0524*P*)^2^ + 0.1317*P*] where *P* = (*F*_o_^2^ + 2*F*_c_^2^)/3
2533 reflections	(Δ/σ)_max_ < 0.001
174 parameters	Δρ_max_ = 0.32 e Å^−^^3^
4 restraints	Δρ_min_ = −0.31 e Å^−^^3^

### Special details

**Table:**

Geometry. All esds (except the esd in the dihedral angle between two l.s. planes) are estimated using the full covariance matrix. The cell esds are taken into account individually in the estimation of esds in distances, angles and torsion angles; correlations between esds in cell parameters are only used when they are defined by crystal symmetry. An approximate (isotropic) treatment of cell esds is used for estimating esds involving l.s. planes.
Refinement. Refinement of F^2^ against ALL reflections. The weighted R-factor wR and goodness of fit S are based on F^2^, conventional R-factors R are based on F, with F set to zero for negative F^2^. The threshold expression of F^2^ > 2sigma(F^2^) is used only for calculating R-factors(gt) etc. and is not relevant to the choice of reflections for refinement. R-factors based on F^2^ are statistically about twice as large as those based on F, and R- factors based on ALL data will be even larger.

### Fractional atomic coordinates and isotropic or equivalent isotropic displacement parameters (Å^2^)

**Table:**

	*x*	*y*	*z*	*U*_iso_*/*U*_eq_	
O1	0.42086 (18)	0.73704 (16)	0.82496 (11)	0.0228 (3)	
O2	0.52698 (19)	0.97324 (17)	0.68889 (11)	0.0249 (3)	
O3	0.0077 (2)	0.36444 (17)	0.67272 (12)	0.0217 (3)	
H31	0.006 (3)	0.245 (2)	0.697 (2)	0.036 (6)*	
H32	−0.104 (4)	0.434 (5)	0.646 (3)	0.099 (11)*	
O4	0.3036 (2)	0.39114 (18)	0.43791 (12)	0.0254 (3)	
H41	0.218 (5)	0.370 (6)	0.517 (2)	0.148 (17)*	
H42	0.339 (4)	0.292 (3)	0.400 (2)	0.051 (7)*	
N1	0.4333 (2)	0.90831 (19)	0.79926 (13)	0.0182 (3)	
N2	0.4577 (2)	1.3147 (2)	0.76156 (15)	0.0218 (3)	
H21	0.466 (3)	1.434 (3)	0.7566 (18)	0.027 (5)*	
H22	0.521 (3)	1.251 (3)	0.698 (2)	0.031 (6)*	
N3	0.0180 (2)	1.01263 (18)	1.24009 (12)	0.0167 (3)	
N4	0.0340 (2)	1.32261 (19)	1.20831 (13)	0.0164 (3)	
H4	0.013 (3)	1.445 (3)	1.231 (2)	0.040 (6)*	
C1	0.3378 (2)	1.0322 (2)	0.89745 (15)	0.0158 (3)	
C2	0.2319 (2)	0.9471 (2)	1.01679 (15)	0.0156 (3)	
H2	0.2269	0.8178	1.0293	0.019*	
C3	0.1361 (2)	1.0585 (2)	1.11424 (15)	0.0148 (3)	
C4	0.1479 (2)	1.2541 (2)	1.09322 (15)	0.0149 (3)	
C5	0.2526 (2)	1.3396 (2)	0.97763 (15)	0.0162 (3)	
H5	0.2572	1.4686	0.9674	0.019*	
C6	0.3532 (2)	1.2294 (2)	0.87459 (15)	0.0163 (3)	
C7	−0.0386 (2)	1.1733 (2)	1.29146 (15)	0.0157 (3)	
C8	−0.1690 (3)	1.1990 (2)	1.42471 (15)	0.0206 (4)	
H8A	−0.2829	1.3129	1.4215	0.031*	
H8B	−0.0876	1.2148	1.4752	0.031*	
H8C	−0.2210	1.0861	1.4637	0.031*	

### Atomic displacement parameters (Å^2^)

**Table:**

	*U*^11^	*U*^22^	*U*^33^	*U*^12^	*U*^13^	*U*^23^
O1	0.0276 (7)	0.0150 (6)	0.0264 (6)	−0.0065 (5)	−0.0035 (5)	−0.0080 (5)
O2	0.0287 (7)	0.0235 (6)	0.0194 (6)	−0.0106 (5)	0.0037 (5)	−0.0061 (5)
O3	0.0281 (7)	0.0135 (6)	0.0253 (6)	−0.0070 (5)	−0.0066 (5)	−0.0045 (5)
O4	0.0267 (7)	0.0215 (7)	0.0261 (7)	−0.0046 (5)	−0.0012 (6)	−0.0099 (5)
N1	0.0168 (7)	0.0178 (7)	0.0215 (7)	−0.0041 (5)	−0.0047 (6)	−0.0066 (5)
N2	0.0257 (8)	0.0174 (7)	0.0207 (8)	−0.0083 (6)	−0.0001 (6)	−0.0043 (6)
N3	0.0174 (7)	0.0154 (7)	0.0180 (7)	−0.0042 (5)	−0.0042 (6)	−0.0046 (5)
N4	0.0191 (7)	0.0129 (7)	0.0175 (7)	−0.0041 (5)	−0.0038 (6)	−0.0043 (5)
C1	0.0147 (8)	0.0156 (8)	0.0180 (8)	−0.0023 (6)	−0.0047 (6)	−0.0061 (6)
C2	0.0151 (8)	0.0131 (7)	0.0210 (8)	−0.0038 (6)	−0.0064 (6)	−0.0045 (6)
C3	0.0139 (8)	0.0147 (7)	0.0175 (8)	−0.0043 (6)	−0.0063 (6)	−0.0021 (6)
C4	0.0140 (8)	0.0142 (7)	0.0186 (8)	−0.0027 (6)	−0.0066 (6)	−0.0043 (6)
C5	0.0177 (8)	0.0112 (7)	0.0211 (8)	−0.0039 (6)	−0.0067 (7)	−0.0025 (6)
C6	0.0140 (8)	0.0175 (8)	0.0185 (8)	−0.0038 (6)	−0.0062 (6)	−0.0022 (6)
C7	0.0158 (8)	0.0146 (7)	0.0187 (8)	−0.0044 (6)	−0.0065 (6)	−0.0031 (6)
C8	0.0239 (9)	0.0182 (8)	0.0191 (8)	−0.0051 (7)	−0.0034 (7)	−0.0053 (6)

### Geometric parameters (Å, °)

**Table:**

O1—N1	1.2383 (17)	C2—C1	1.401 (2)
O2—N1	1.2487 (17)	C2—C3	1.364 (2)
O3—H31	0.854 (16)	C2—H2	0.9300
O3—H32	0.890 (18)	C4—N4	1.376 (2)
O4—H41	0.899 (19)	C4—C3	1.414 (2)
O4—H42	0.845 (16)	C4—C5	1.372 (2)
N1—C1	1.429 (2)	C5—C6	1.409 (2)
N2—C6	1.352 (2)	C5—H5	0.9300
N2—H21	0.87 (2)	C6—C1	1.433 (2)
N2—H22	0.85 (2)	C8—C7	1.482 (2)
N3—C3	1.398 (2)	C8—H8A	0.9600
N3—C7	1.310 (2)	C8—H8B	0.9600
N4—C7	1.370 (2)	C8—H8C	0.9600
N4—H4	0.94 (2)		
			
H31—O3—H32	111 (3)	C2—C3—C4	119.69 (14)
H42—O4—H41	110 (3)	N4—C4—C3	104.44 (13)
O1—N1—O2	120.75 (13)	C5—C4—N4	132.73 (14)
O1—N1—C1	119.17 (13)	C5—C4—C3	122.82 (14)
O2—N1—C1	120.08 (13)	C4—C5—C6	119.18 (14)
C6—N2—H21	118.8 (13)	C4—C5—H5	120.4
C6—N2—H22	120.7 (14)	C6—C5—H5	120.4
H21—N2—H22	120.4 (19)	N2—C6—C1	124.24 (15)
C7—N3—C3	104.78 (13)	N2—C6—C5	118.63 (14)
C4—N4—H4	128.3 (13)	C5—C6—C1	117.13 (14)
C7—N4—C4	107.67 (13)	N3—C7—N4	113.09 (14)
C7—N4—H4	123.9 (13)	N3—C7—C8	125.40 (14)
N1—C1—C6	121.60 (14)	N4—C7—C8	121.52 (14)
C2—C1—N1	115.68 (14)	C7—C8—H8A	109.5
C2—C1—C6	122.71 (14)	C7—C8—H8B	109.5
C1—C2—H2	120.8	C7—C8—H8C	109.5
C3—C2—C1	118.45 (14)	H8A—C8—H8B	109.5
C3—C2—H2	120.8	H8A—C8—H8C	109.5
N3—C3—C4	110.02 (13)	H8B—C8—H8C	109.5
C2—C3—N3	130.29 (14)		
			
O1—N1—C1—C2	−1.3 (2)	C3—C4—N4—C7	0.29 (16)
O1—N1—C1—C6	177.83 (14)	C5—C4—N4—C7	179.23 (16)
O2—N1—C1—C2	178.33 (14)	N4—C4—C3—C2	179.06 (13)
O2—N1—C1—C6	−2.6 (2)	N4—C4—C3—N3	−0.32 (17)
C7—N3—C3—C2	−179.07 (16)	C5—C4—C3—N3	−179.40 (14)
C7—N3—C3—C4	0.23 (17)	C5—C4—C3—C2	0.0 (2)
C3—N3—C7—N4	−0.05 (17)	N4—C4—C5—C6	−178.66 (16)
C3—N3—C7—C8	179.45 (15)	C3—C4—C5—C6	0.1 (2)
C4—N4—C7—N3	−0.16 (18)	C4—C5—C6—N2	−179.35 (14)
C4—N4—C7—C8	−179.68 (14)	C4—C5—C6—C1	0.5 (2)
C3—C2—C1—N1	−179.56 (13)	N2—C6—C1—N1	−0.4 (2)
C3—C2—C1—C6	1.3 (2)	N2—C6—C1—C2	178.59 (15)
C1—C2—C3—N3	178.54 (15)	C5—C6—C1—N1	179.73 (14)
C1—C2—C3—C4	−0.7 (2)	C5—C6—C1—C2	−1.2 (2)

### Hydrogen-bond geometry (Å, °)

**Table:**

*D*—H···*A*	*D*—H	H···*A*	*D*···*A*	*D*—H···*A*
N4—H4···O3^i^	0.94 (2)	1.87 (2)	2.7735 (18)	160.4 (19)
N2—H21···O1^ii^	0.88 (2)	2.39 (2)	3.2212 (18)	158.8 (17)
N2—H21···O4^iii^	0.88 (2)	2.59 (2)	3.163 (2)	124.1 (15)
N2—H22···O2	0.85 (2)	2.03 (2)	2.6387 (19)	127.3 (19)
O3—H31···N3^iv^	0.85 (2)	1.89 (2)	2.7354 (18)	176.(2)
O3—H32···O4^v^	0.89 (3)	1.90 (3)	2.776 (2)	168 (3)
O4—H41···O3	0.90 (2)	1.88 (3)	2.7727 (19)	170 (4)
O4—H42···O1^vi^	0.85 (2)	2.53 (2)	3.0930 (17)	125.(2)
O4—H42···O2^vi^	0.85 (2)	2.17 (2)	3.0126 (17)	171 (3)
C5—H5···O1^ii^	0.93	2.54	3.3556 (19)	146

Symmetry codes: (i) −*x*, −*y*+2, −*z*+2; (ii) *x*, *y*+1, *z*; (iii) −*x*+1, −*y*+2, −*z*+1; (iv) −*x*, −*y*+1, −*z*+2; (v) −*x*, −*y*+1, −*z*+1; (vi) −*x*+1, −*y*+1, −*z*+1.
